# Development and characterization of microsatellite loci for common raven (*Corvus corax*) and cross species amplification in other Corvidae

**DOI:** 10.1186/s13104-015-1643-5

**Published:** 2015-11-06

**Authors:** Christin L. Pruett, Leping Wan, Tianyu Li, Cory Spern, Stacey L. Lance, Travis Glenn, Brant Faircloth, Kevin Winker

**Affiliations:** Department of Biological Sciences, Florida Institute of Technology, Melbourne, FL 32901 USA; Savannah River Ecology Laboratory, University of Georgia, Aiken, SC 29802 USA; Department of Environmental Health Science, University of Georgia, Athens, GA 30602 USA; Department of Biological Sciences, Louisiana State University, Baton Rouge, LA 70803 USA; University of Alaska Museum, University of Alaska Fairbanks, Fairbanks, AK 99775 USA

**Keywords:** *Corvus*, PCR primers, Microsatellite, Holarctic, Alaska

## Abstract

**Background:**

A priority for conservation is the identification of endemic populations. We developed microsatellite markers for common raven (*Corvus corax*), a bird species with a Holarctic distribution, to identify and assess endemic populations in Alaska.

**Results:**

From a total of 50 microsatellite loci, we isolated and characterized 15 loci. Eight of these loci were polymorphic and readily scoreable. Eighteen to 20 common ravens from Fairbanks, Alaska were genotyped showing the following variability: 3–8 alleles per locus, 0.25–0.80 observed heterozygosity (H_o_), and 0.30–0.80 expected heterozygosity (H_e_). All loci were in Hardy–Weinberg equilibrium and linkage equilibrium and many loci amplified and were polymorphic in related taxa.

**Conclusions:**

These loci will be used to identify endemic populations of common raven and assess their genetic diversity and connectivity.

## Findings

Common raven (*Corvus corax*) is a widespread, Holarctic bird that is listed as least concern on the IUCN Red List of Threatened Species (http://www.iucnredlist.org/search). Although common ravens in Alaska are not morphologically diverse (2 subspecies) [1], genetic assessments using mitochondrial DNA markers have shown differences among lineages [2] and have suggested that isolated, endemic populations are found in the Aleutian Islands of Alaska [3]. Island populations were possibly isolated in glacial refugia as has been suggested in other studies of Aleutian landbirds [3–6]. Alternatively, populations might be post-glacial in origin having colonized after the last glacial maximum (~15,000 ybp) [7]. Given the recent timing of these events, a quickly evolving molecular marker is needed to assess the genetic diversity of populations, connectivity among populations, and to understand the history of ravens throughout the Holarctic. We developed eight microsatellite loci that will be used to assess population structure, rates of gene flow, colonization history, and to identify isolated populations of common raven.

Total genomic DNA was extracted from the muscle tissue of two common ravens from Alaska using a QiAamp DNA Mini Kit (Qiagen, Valencia, CA). The protocols of Glenn and Schable [8] and Katzinel et al. [9] were used to isolate microsatellite loci. Briefly, restriction enzymes were used to digest DNA, adapters were ligated onto fragments, microsatellite-containing fragments were separated using biotinylated probes and streptavidin beads, and fragments yields were increased using PCR. Fragments were run on a Roche 454 FLX with Titanium chemistry (454 Life Sciences, a Roche Company, Branford, CT) with similarly treated libraries, each with combinations of unique adapter and Roche MID-tags. We used demuxipy (https://github.com/faircloth-lab/demuxipy/) to demultiplex fragments, and used a version of MSATCOMMANDER [10] to find 591 sequences containing microsatellites from 1467 sequence reads. Primer 3 [11] was used to design primers with the CAG primer sequence (5′-CAGTCGGGCGTCATCA-3′) prepended to the 5′ end of one primer of each pair. This enables the use of a fluorescently labeled primer with the CAG sequence during PCR to tag amplified fragments [12, 13]. A short sequence (GTTT) was prepended to the primer without the CAG sequence to encourage adenylation of the amplicons for accurate genotyping [14, 15].

Fifty primer pairs were tested for amplification and polymorphism using the DNA from 10 individuals. Amplifications were in 20 µl volumes [250 µg/mL BSA, 10× Buffer B (Fisher Scientific), 25 mM MgCl_2_, 5 µM unlabeled primer, 0.5 µM tag labeled primer, 5 µM universal dye-labeled primer, 2.5 mM dNTPs, 0.5 units Taq DNA polymerase (Fisher Scientific), and 20 ng DNA template] using a BioRad MyCycler thermal cycler. Touchdown cycling conditions were used to amplify DNA and for attachment of the universal dye-labeled primer with the CAG tag. PCR parameters included an initial denaturation step of 2 min 30 s at 95 °C then 20 cycles of 95 °C for 20 s, 65 °C to 50 °C annealing temperature for 20 s (decreasing 0.5 °C per cycle), and extension step of 72 °C for 30 s followed by 15 cycles of 95 °C for 20 s, 55 °C to 45 °C for 20 s, and 72 °C for 30 s. Cycles were followed with a final extension step of 72 °C for 10 min. An ABI3730XL sequencer (Applied Biosystems) was used to determine genotypes. We found that fifteen primers amplified a product and eight of these primers exhibited polymorphism.

Initially, we tested for polymorphism using ten individuals from seven locations in Alaska including locations from the Aleutian Islands, the mainland of Alaska, and islands in southeast Alaska. Herein, we report the statistics associated with polymorphic loci using 18–20 individuals from Fairbanks, Alaska to avoid any Wahlund effect caused by population structure [16]. We also tested the polymorphism of one to three individuals each for seven other species in the family Corvidae (Tables 1, 2) to assess cross-species amplification. We used Gene Mapper software (Applied Biosystems) to score alleles and to determine the number of alleles per locus (K), observed heterozygosity (H_o_), and expected heterozygosity (H_e_), using Arlequin ver. 3.5 [17]. We also tested for Hardy–Weinberg equilibrium and linkage disequilibrium using Arlequin and used Microchecker [18] to test for genotyping errors. We found that the number of alleles per locus ranged from 3 to 8, H_o_ ranged from 0.25 to 0.80, and H_e_ ranged from 0.30 to 0.80 for common ravens (Table 1). All loci were in Hardy–Weinberg equilibrium and linkage equilibrium after correction for multiple tests and we found no evidence of null alleles, stuttering, or large-allele dropout.

**Table 1:** Characteristics of eight microsatellite loci in the common raven *Corvus corax*

Locus	Primer sequence (5′–3′)	Primer label	Repeat motif		T_a_ (°C)	n	HWE	K	H_o_	H_e_	GenBank accession no.
Coco6	F: *AACCAAGCTGTCAAAGGAGA	VIC	(ATCT)^13^		60/50	20	0.396	8	0.55	0.54	KT288258
	R: TTTCCTGGTGCGGAGAAGTA										
Coco12	F: *GAGGCTTTCAGCGATATGGG	6FAM	(AGAT)^7^		60/50	18	0.173	5	0.72	0.72	KT288259
	R: GCTGCACTTGCCTGTTGTTTA										
Coco30	F: *GTGCAAACCACTCCAGAAT	6FAM	(GGAT)^16^		60/50	20	0.910	6	0.70	0.80	KT288260
	R: GCTGTCACCAGTGGCTTTAAT										
Coco31	F: *TGGCTACTTACCCGGAATCTC	NED	(ATGT)^8^		60/50	20	0.470	3	0.25	0.30	KT288261
	R: CTTCTCTACCGCCCATCACA										
Coco32	F: ATGCAAATCTCCGTGTATCA	NED	(ATCT)^9^		60/50	20	0.422	8	0.80	0.80	KT288262
	R: *CCTCCCTTTGTCCTTTCTGG										
Coco36	F: CCTTCTGCGCTGTGTTCAAA	NED	(CTT)^8^		60/50	20	0.008	5	0.40	0.71	KT288263
	R: *CTGAGGTGCAAGCTTTCCTG										
Coco45	F: TGACCAACACAGAGCAGATATT	6FAM	(GT)^11^		60/50	19	0.353	3	0.53	0.54	KT288264
	R: *GGAACCTCTGAGTCCAAA										
Coco50	F: *ATCCCATCAAAGGTCCACG	VIC	(AC)^13^		60/50	20	0.157	5	0.50	0.64	KT288265
	R: CCACGACAGCTGATGAGAGA										

All loci were in Hardy–Weinberg and linkage equilibrium after Bonferroni correction for multiple tests

*Asterisks* show location of CAG tag for each primer pair, *T*_*a*_ annealing temperatures for touchdown protocol for each locus, *n* number of individuals genotyped, *K* number of alleles, *H*_*o*_ observed heterozygosity, *H*_*e*_ expected heterozygosity

**Table 2:** Number of alleles in eight microsatellite loci in seven Corvidae species

Species	n	Number of alleles per locus							
		Coco6	Coco12	Coco30	Coco31	Coco32	Coco36	Coco45	Coco50
Fish crow (*Corvus ossifragus*)	2	4	1	3	1	3	2	2	1
Northwestern crow (*Corvus caurinus*)	2	–	–	3	1	1	3	3	1
American crow (*Corvus brachyrhynchos*)	1	–	–	2	2	1	2	1	1
Steller’s jay (*Cyanocitta stelleri*)	3	–	–	2	1	–	2	1	3
Blue jay (*Cyanocitta cristata*)	2	–	–	3	2	–	2	1	2
Gray jay (*Perisoreus canadensis*)	2	–	–	3	1	4	1	1	2
Florida scrub-jay (*Aphelocoma coerulescens*)	3	–	–	–	–	–	–	–	–

*n* number of individuals attempted to amplify and genotype, – individuals failed to amplify at locus

Preliminary data from nine Alaska populations of common raven showed that the loci developed in this study are useful for population-level assessments with pairwise FST values ranging from 0.011 to 0.51. In addition, these loci are polymorphic in these populations (H_e_ 0.40–0.74; alleles per locus, 2.88–6.75). Eight loci that were developed for other Corvidae have shown polymorphism in common raven [19]. By combining our newly developed markers with those from other taxa, it is possible that researchers could assess very fine-scale population structure.

In other Corvidae taxa, we found that the majority of loci amplified and were polymorphic in six of the seven species (Table 2). All loci amplified in the fish crow (*Corvus ossifragus*) and five of eight loci were polymorphic. Loci that were monomorphic in other taxa might be polymorphic if a larger sample size was assessed. We suggest that these loci could be successfully used for population-level assessments in these species (Table 2) and possibly in other members of the genera *Corvus* and *Perisoreus* but are unlikely to be useful in *Aphelocoma* (Table 2).

## Availability of the supporting data

Microsatellite sequences were deposited in the National Center for Biotechnology Information (http://www.ncbi.nlm.nih.gov). They are accessible via GenBank accession numbers listed in Table 1.
